# 1,4-Bis(4-*tert*-but­ylbenz­yl)piperazine

**DOI:** 10.1107/S1600536811044114

**Published:** 2011-10-29

**Authors:** Li-Juan Luo, Jian-Quan Weng

**Affiliations:** a Department of Chemical Engineering, Ningbo University of Technology, Ningbo 315016, People’s Republic of China; b College of Chemical Engineering and Materials Science, Zhejiang University of Technology, Hangzhou 310014, People’s Republic of China

## Abstract

The complete mol­ecule of the title compound, C_26_H_38_N_2_, is generated by a crystallographic inversion centre. The piperazine ring adopts a chair conformation with pseudo-equatorial substituents. In the crystal, mol­ecules inter­act only by van der Waals forces.

## Related literature

For related structures, see: Ma *et al.* (2007 ▶); Liu *et al.* (2011 ▶).
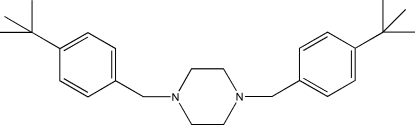


## Experimental

### 

#### Crystal data

C_26_H_38_N_2_
*M* *_r_* = 378.58
Triclinic, 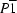

*a* = 6.162 (4) Å
*b* = 9.616 (5) Å
*c* = 10.656 (7) Å
α = 114.279 (19)°
β = 92.42 (5)°
γ = 96.50 (4)°
*V* = 569.1 (6) Å^3^
*Z* = 1
Mo *K*α radiation
μ = 0.06 mm^−1^
*T* = 113 K
0.24 × 0.20 × 0.08 mm

#### Data collection

Rigaku Saturn724 CCD diffractometer
Absorption correction: multi-scan (*CrystalClear*; Rigaku/MSC, 2005 ▶) *T*_min_ = 0.985, *T*_max_ = 0.995
6003 measured reflections
2686 independent reflections
1481 reflections with *I* > 2σ(*I*)
*R*_int_ = 0.041

#### Refinement

*R*[*F* ^2^ > 2σ(*F* ^2^)] = 0.041
*wR*(*F* ^2^) = 0.099
*S* = 1.01
2686 reflections
130 parameters
H-atom parameters constrained
Δρ_max_ = 0.16 e Å^−3^
Δρ_min_ = −0.18 e Å^−3^

### 

Data collection: *CrystalClear* (Rigaku/MSC, 2005 ▶); cell refinement: *CrystalClear*; data reduction: *CrystalClear*; program(s) used to solve structure: *SHELXS97* (Sheldrick, 2008 ▶); program(s) used to refine structure: *SHELXL97* (Sheldrick, 2008 ▶); molecular graphics: *SHELXTL* (Sheldrick, 2008 ▶); software used to prepare material for publication: *CrystalStructure* (Rigaku/MSC, 2005 ▶).

## Supplementary Material

Crystal structure: contains datablock(s) global, I. DOI: 10.1107/S1600536811044114/hb6451sup1.cif

Structure factors: contains datablock(s) I. DOI: 10.1107/S1600536811044114/hb6451Isup2.hkl

Supplementary material file. DOI: 10.1107/S1600536811044114/hb6451Isup3.cml

Additional supplementary materials:  crystallographic information; 3D view; checkCIF report

## Figures and Tables

**Fig. 1.**
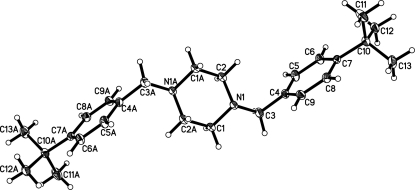
The molecular structure of (I). Displacement ellipsoids are drawn at the 30% probability level and H atoms are shown as small spheres of arbitrary radius.

**Fig. 2.**
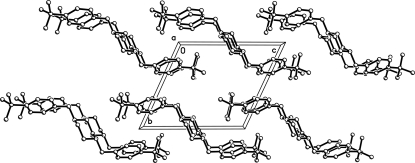
The crystal packing for (I).

## supplementary crystallographic information

### Experimental

Piperazine (50 mmol), dissolved in 20 ml 96% of ethanol, was added dropwise to a stirred solution of *tert*-butyl benzyl (50 mmol) at reflux. The mixture was stirred for 8 h at reflux, TLC monitored. The mixture was stirred overnight at room temperature, evaporated in vacuum and the residue was purified by recrystallization from ethanol to give the title compound, (I). Colourless prisms of (I) were grown from ethanol.

### Refinement

All the H atoms were positioned geometrically (C—H = 0.93–0.97 Å) and refined as riding with *U*_iso_(H) = 1.2*U*_eq_(C) or 1.5*U*_eq_(methyl C).

### Crystal data

**Table:**

C_26_H_38_N_2_	*Z* = 1
*M**_r_* = 378.58	*F*(000) = 208
Triclinic, *P*1	*D*_x_ = 1.105 Mg m^−^^3^
Hall symbol: -P 1	Mo *K*α radiation, λ = 0.71073 Å
*a* = 6.162 (4) Å	Cell parameters from 1980 reflections
*b* = 9.616 (5) Å	θ = 2.1–27.9°
*c* = 10.656 (7) Å	µ = 0.06 mm^−^^1^
α = 114.279 (19)°	*T* = 113 K
β = 92.42 (5)°	Prism, colorless
γ = 96.50 (4)°	0.24 × 0.20 × 0.08 mm
*V* = 569.1 (6) Å^3^	

### Data collection

**Table:**

Rigaku Saturn724 CCD diffractometer	2686 independent reflections
Radiation source: rotating anode	1481 reflections with *I* > 2σ(*I*)
multilayer	*R*_int_ = 0.041
Detector resolution: 14.22 pixels mm^-1^	θ_max_ = 27.9°, θ_min_ = 2.1°
ω and φ scans	*h* = −8→7
Absorption correction: multi-scan (*CrystalClear*; Rigaku/MSC, 2005)	*k* = −12→12
*T*_min_ = 0.985, *T*_max_ = 0.995	*l* = −13→14
6003 measured reflections	

### Refinement

**Table:**

Refinement on *F*^2^	Primary atom site location: structure-invariant direct methods
Least-squares matrix: full	Secondary atom site location: difference Fourier map
*R*[*F*^2^ > 2σ(*F*^2^)] = 0.041	Hydrogen site location: inferred from neighbouring sites
*wR*(*F*^2^) = 0.099	H-atom parameters constrained
*S* = 1.01	*w* = 1/[σ^2^(*F*_o_^2^) + (0.034*P*)^2^] where *P* = (*F*_o_^2^ + 2*F*_c_^2^)/3
2686 reflections	(Δ/σ)_max_ < 0.001
130 parameters	Δρ_max_ = 0.16 e Å^−^^3^
0 restraints	Δρ_min_ = −0.18 e Å^−^^3^

### Special details

**Table:**

Geometry. All e.s.d.'s (except the e.s.d. in the dihedral angle between two l.s. planes) are estimated using the full covariance matrix. The cell e.s.d.'s are taken into account individually in the estimation of e.s.d.'s in distances, angles and torsion angles; correlations between e.s.d.'s in cell parameters are only used when they are defined by crystal symmetry. An approximate (isotropic) treatment of cell e.s.d.'s is used for estimating e.s.d.'s involving l.s. planes.
Refinement. Refinement of *F*^2^ against ALL reflections. The weighted *R*-factor *wR* and goodness of fit *S* are based on *F*^2^, conventional *R*-factors *R* are based on *F*, with *F* set to zero for negative *F*^2^. The threshold expression of *F*^2^ > σ(*F*^2^) is used only for calculating *R*-factors(gt) *etc*. and is not relevant to the choice of reflections for refinement. *R*-factors based on *F*^2^ are statistically about twice as large as those based on *F*, and *R*- factors based on ALL data will be even larger.

### Fractional atomic coordinates and isotropic or equivalent isotropic displacement parameters (Å^2^)

**Table:**

	*x*	*y*	*z*	*U*_iso_*/*U*_eq_	
N1	1.04698 (14)	0.10740 (11)	0.64238 (9)	0.0276 (3)	
C1	1.19772 (18)	0.10329 (14)	0.53912 (12)	0.0304 (3)	
H1A	1.3503	0.1354	0.5835	0.036*	
H1B	1.1644	0.1765	0.4999	0.036*	
C2	0.82344 (17)	0.05712 (14)	0.57527 (11)	0.0296 (3)	
H2A	0.7838	0.1295	0.5366	0.036*	
H2B	0.7205	0.0582	0.6444	0.036*	
C3	1.0687 (2)	0.26252 (14)	0.75487 (12)	0.0349 (3)	
H3A	1.0114	0.3328	0.7192	0.042*	
H3B	1.2260	0.3006	0.7869	0.042*	
C4	0.94701 (19)	0.26622 (13)	0.87580 (11)	0.0283 (3)	
C5	0.7673 (2)	0.34155 (14)	0.91236 (12)	0.0354 (3)	
H5	0.7138	0.3899	0.8579	0.042*	
C6	0.66197 (19)	0.34862 (14)	1.02736 (12)	0.0325 (3)	
H6	0.5377	0.4012	1.0492	0.039*	
C7	0.73370 (17)	0.28090 (12)	1.11086 (11)	0.0244 (3)	
C8	0.91275 (17)	0.20106 (13)	1.07092 (12)	0.0301 (3)	
H8	0.9643	0.1500	1.1235	0.036*	
C9	1.01657 (18)	0.19463 (14)	0.95659 (12)	0.0316 (3)	
H9	1.1385	0.1398	0.9328	0.038*	
C10	0.62717 (18)	0.28918 (14)	1.24015 (12)	0.0292 (3)	
C11	0.5127 (2)	0.12960 (15)	1.21512 (15)	0.0519 (4)	
H11A	0.4028	0.0923	1.1350	0.078*	
H11B	0.4404	0.1356	1.2970	0.078*	
H11C	0.6212	0.0584	1.1973	0.078*	
C12	0.45875 (19)	0.40197 (14)	1.28026 (12)	0.0360 (3)	
H12A	0.5309	0.5053	1.2976	0.054*	
H12B	0.3964	0.4045	1.3641	0.054*	
H12C	0.3413	0.3683	1.2047	0.054*	
C13	0.8046 (2)	0.34597 (17)	1.36335 (12)	0.0448 (4)	
H13A	0.9099	0.2723	1.3441	0.067*	
H13B	0.7356	0.3547	1.4469	0.067*	
H13C	0.8814	0.4470	1.3774	0.067*	

### Atomic displacement parameters (Å^2^)

**Table:**

	*U*^11^	*U*^22^	*U*^33^	*U*^12^	*U*^13^	*U*^23^
N1	0.0278 (5)	0.0292 (6)	0.0238 (5)	0.0025 (4)	0.0087 (4)	0.0089 (5)
C1	0.0275 (6)	0.0365 (8)	0.0272 (6)	0.0022 (5)	0.0094 (5)	0.0132 (6)
C2	0.0304 (7)	0.0347 (7)	0.0274 (6)	0.0078 (5)	0.0107 (5)	0.0151 (6)
C3	0.0433 (7)	0.0305 (7)	0.0271 (7)	−0.0004 (6)	0.0118 (6)	0.0090 (6)
C4	0.0344 (7)	0.0234 (6)	0.0229 (6)	−0.0004 (5)	0.0076 (5)	0.0061 (5)
C5	0.0478 (8)	0.0366 (8)	0.0275 (7)	0.0138 (6)	0.0079 (6)	0.0167 (6)
C6	0.0359 (7)	0.0354 (7)	0.0291 (7)	0.0147 (6)	0.0092 (5)	0.0134 (6)
C7	0.0269 (6)	0.0216 (6)	0.0204 (6)	0.0006 (5)	0.0029 (5)	0.0051 (5)
C8	0.0328 (7)	0.0318 (7)	0.0276 (6)	0.0060 (5)	0.0027 (5)	0.0139 (6)
C9	0.0297 (7)	0.0330 (7)	0.0312 (7)	0.0079 (5)	0.0099 (6)	0.0111 (6)
C10	0.0345 (7)	0.0302 (7)	0.0242 (6)	0.0061 (5)	0.0088 (5)	0.0118 (5)
C11	0.0698 (10)	0.0356 (8)	0.0532 (9)	0.0059 (7)	0.0367 (8)	0.0192 (7)
C12	0.0390 (7)	0.0395 (8)	0.0278 (7)	0.0099 (6)	0.0111 (6)	0.0105 (6)
C13	0.0510 (8)	0.0611 (10)	0.0255 (7)	0.0164 (7)	0.0073 (6)	0.0190 (7)

### Geometric parameters (Å, °)

**Table:**

N1—C2	1.4575 (17)	C7—C8	1.3968 (16)
N1—C1	1.4609 (17)	C7—C10	1.5275 (18)
N1—C3	1.4665 (16)	C8—C9	1.3821 (17)
C1—C2^i^	1.5089 (17)	C8—H8	0.9500
C1—H1A	0.9900	C9—H9	0.9500
C1—H1B	0.9900	C10—C11	1.5251 (19)
C2—C1^i^	1.5090 (17)	C10—C12	1.5321 (17)
C2—H2A	0.9900	C10—C13	1.540 (2)
C2—H2B	0.9900	C11—H11A	0.9800
C3—C4	1.5079 (18)	C11—H11B	0.9800
C3—H3A	0.9900	C11—H11C	0.9800
C3—H3B	0.9900	C12—H12A	0.9800
C4—C5	1.3742 (17)	C12—H12B	0.9800
C4—C9	1.3863 (17)	C12—H12C	0.9800
C5—C6	1.3916 (18)	C13—H13A	0.9800
C5—H5	0.9500	C13—H13B	0.9800
C6—C7	1.3855 (17)	C13—H13C	0.9800
C6—H6	0.9500		
			
C2—N1—C1	109.05 (10)	C8—C7—C10	119.97 (11)
C2—N1—C3	111.16 (11)	C9—C8—C7	121.48 (12)
C1—N1—C3	110.69 (10)	C9—C8—H8	119.3
N1—C1—C2^i^	110.36 (10)	C7—C8—H8	119.3
N1—C1—H1A	109.6	C8—C9—C4	121.58 (11)
C2^i^—C1—H1A	109.6	C8—C9—H9	119.2
N1—C1—H1B	109.6	C4—C9—H9	119.2
C2^i^—C1—H1B	109.6	C11—C10—C7	109.50 (10)
H1A—C1—H1B	108.1	C11—C10—C12	108.72 (11)
N1—C2—C1^i^	110.74 (11)	C7—C10—C12	112.36 (11)
N1—C2—H2A	109.5	C11—C10—C13	109.56 (12)
C1^i^—C2—H2A	109.5	C7—C10—C13	109.51 (10)
N1—C2—H2B	109.5	C12—C10—C13	107.14 (11)
C1^i^—C2—H2B	109.5	C10—C11—H11A	109.5
H2A—C2—H2B	108.1	C10—C11—H11B	109.5
N1—C3—C4	112.51 (11)	H11A—C11—H11B	109.5
N1—C3—H3A	109.1	C10—C11—H11C	109.5
C4—C3—H3A	109.1	H11A—C11—H11C	109.5
N1—C3—H3B	109.1	H11B—C11—H11C	109.5
C4—C3—H3B	109.1	C10—C12—H12A	109.5
H3A—C3—H3B	107.8	C10—C12—H12B	109.5
C5—C4—C9	117.25 (11)	H12A—C12—H12B	109.5
C5—C4—C3	122.29 (12)	C10—C12—H12C	109.5
C9—C4—C3	120.46 (11)	H12A—C12—H12C	109.5
C4—C5—C6	121.51 (12)	H12B—C12—H12C	109.5
C4—C5—H5	119.2	C10—C13—H13A	109.5
C6—C5—H5	119.2	C10—C13—H13B	109.5
C7—C6—C5	121.68 (11)	H13A—C13—H13B	109.5
C7—C6—H6	119.2	C10—C13—H13C	109.5
C5—C6—H6	119.2	H13A—C13—H13C	109.5
C6—C7—C8	116.46 (11)	H13B—C13—H13C	109.5
C6—C7—C10	123.57 (11)		
			
C2—N1—C1—C2^i^	−58.15 (14)	C5—C6—C7—C10	178.35 (10)
C3—N1—C1—C2^i^	179.26 (9)	C6—C7—C8—C9	2.03 (16)
C1—N1—C2—C1^i^	58.37 (14)	C10—C7—C8—C9	−178.31 (10)
C3—N1—C2—C1^i^	−179.32 (10)	C7—C8—C9—C4	−0.42 (18)
C2—N1—C3—C4	68.92 (14)	C5—C4—C9—C8	−1.27 (17)
C1—N1—C3—C4	−169.72 (9)	C3—C4—C9—C8	177.64 (10)
N1—C3—C4—C5	−113.26 (14)	C6—C7—C10—C11	111.43 (14)
N1—C3—C4—C9	67.89 (15)	C8—C7—C10—C11	−68.21 (14)
C9—C4—C5—C6	1.30 (17)	C6—C7—C10—C12	−9.49 (16)
C3—C4—C5—C6	−177.58 (11)	C8—C7—C10—C12	170.87 (10)
C4—C5—C6—C7	0.36 (19)	C6—C7—C10—C13	−128.42 (13)
C5—C6—C7—C8	−2.00 (17)	C8—C7—C10—C13	51.94 (14)

Symmetry codes: (i) −*x*+2, −*y*, −*z*+1.
